# Growth, Health and Physiological Responses of Freshwater-Reared Atlantic Salmon (*Salmo salar*) Fed Graded Dietary Lipid Levels

**DOI:** 10.3390/ani16030356

**Published:** 2026-01-23

**Authors:** Byoungyoon Lee, Junoh Lee, Saeyeon Lim, Gwanghyeok Kim, Minjae Seong, Dahyun Jeong, Sijun Han, Byung-Hwa Min, Kang-Woong Kim, Seong-Mok Jeong, Mun Chang Park, Woo Seok Hong, Se Ryun Kwon, Youngjin Park

**Affiliations:** 1 Department of Aquatic Life Medical Sciences, Sunmoon University, Asan 31460, Republic of Korea; yoon980912@naver.com (B.L.);; 2 Aquafeed Research Center, National Institute of Fisheries Science (NIFS), Pohang 37517, Republic of Korea; 3 National Institute of Fisheries Science, 22, Jeollanam-do, Yeosu-si 59780, Republic of Korea; 4 Jeju Fisheries Research Institute, National Institute of Fisheries Science, Jeju 63068, Republic of Korea; 5 Gangwon State Inland Water Resource Center, Chuncheon 24210, Republic of Korea; 6 Smart Salmon Research Center, Gangneung 25435, Republic of Korea

**Keywords:** Atlantic salmon, dietary lipid, physiological, metabolism, RNA-seq

## Abstract

This study evaluated the optimal dietary lipid level for Atlantic salmon (*Salmo salar*) reared in freshwater, aiming to provide foundational knowledge for the development of cost-effective and nutritionally balanced aquafeeds. Four experimental diets were formulated to contain comparable crude protein levels (47%) but graded lipid levels of 14% (L14), 16% (L16), 18% (L18), and 20% (L20), and were fed to salmon with an initial mean body weight of 241.5 ± 9.7 g during a 12-week feeding trial. Fish in the L16 group exhibited the highest weight gain (WG) and feed efficiency (FE), whereas those in the L14 group showed significantly reduced growth performance. Antioxidant analysis revealed that glutathione peroxidase (GPx) activity was lowest in the L14 group (*p* < 0.05), while plasma glucose concentration was minimized in the L16 group (*p* < 0.05). Transcriptomic profiling of liver tissue from the L14 and L16 groups identified 2117 differentially expressed genes (DEGs). Genes associated with lipid metabolism were more highly expressed in the L16 group, whereas immune- and inflammation-related genes were upregulated in the L14 group. These findings suggest that a dietary lipid level of approximately 16% is most favorable for promoting growth, metabolic stability, and overall health in freshwater-reared Atlantic salmon, thereby providing practical guidance for optimizing feed formulation and improving the economic efficiency of freshwater salmon aquaculture.

## Simple Summary

Atlantic salmon require large amounts of energy during smoltification and seawater transfer, processes that involve major physiological and metabolic adjustments. To meet these energy demands, high-lipid diets are widely used in Atlantic salmon aquaculture. However, excessive dietary lipid intake has been associated with negative effects, including metabolic stress, abnormal lipid accumulation, and impaired immune responses. In recent years, Atlantic salmon have been increasingly reared entirely in freshwater without undergoing seawater transfer, raising questions about whether high-lipid diets are still necessary under these conditions. In this study, we evaluated the optimal dietary lipid level for Atlantic salmon reared exclusively in freshwater by assessing growth performance, antioxidant capacity, metabolic responses, and liver gene expression. Salmon were fed diets containing graded lipid levels (14–20%) for 12 weeks to evaluate their effects on growth performance, antioxidant enzyme activity, hematological parameters, and gene expression profiles. These findings demonstrate that high dietary lipid levels are not required for freshwater-reared Atlantic salmon. Optimizing lipid inclusion in freshwater salmon feeds may reduce feed costs and support more sustainable salmon aquaculture.

## 1. Introduction

Aquaculture is a critical component of global food resources and continues to grow [1,2,3]. In parallel with this growth, the production and market demand for aquafeeds are also increasing. Aquafeeds serve to supply fish with essential nutrients and are directly linked to their nutritional physiology [4]. Therefore, the use of appropriate feeds is essential to ensure efficient growth and optimal health in aquaculture species [5]. Aquafeeds are formulated with dietary proteins and lipids to provide indispensable amino acids and fatty acids required for normal physiological functions [6]. In recent years, high-energy diets with elevated levels of dietary lipids have become increasingly prevalent, aiming to support faster and more efficient growth performance in cultured fish species [7,8].

Dietary lipids are essential components of formulated aquafeeds, serving as a primary energy source for fish with a higher energy density than proteins or carbohydrates. Lipids play various critical roles in supporting growth performance, body composition, and overall health in aquaculture species [9,10]. In addition, dietary lipids are the sole source of essential fatty acids, which are crucial for maintaining normal physiological functions and immune competence in fish [7,11,12]. However, excessive lipid intake has been associated with adverse effects in various fish species, including abnormal lipid deposition, impaired lipid metabolism, increased physiological stress, inflammation, and hepatic steatosis [13,14,15,16]. Therefore, determining species-specific optimal lipid requirements remains a critical objective in aquaculture nutrition research and continues to be the focus of extensive investigation.

Among aquaculture species, dietary lipid intake and requirements have been extensively studied in Atlantic salmon (*Salmo salar*). This is largely due to their unique migratory life cycle, which includes a smoltification phase during which juveniles undergo significant structural, physiological, and functional transformations in preparation for migration from freshwater to seawater. These changes include alterations in osmoregulatory capacity, increased oxygen consumption, and elevated metabolic rates, all of which necessitate a sufficient energy supply during this critical period [17,18]. Lipids and fatty acids play essential roles in these processes, contributing to osmoregulation, metabolic regulation, membrane structure stabilization, and transmembrane transport [19,20]. Consequently, the intake of adequate levels of essential fatty acids has been identified as a key factor in regulating smoltification in salmonids [12,21,22]. For these reasons, dietary lipids have been considered a critical nutritional component in Atlantic salmon aquaculture, and high-lipid diets are increasingly adopted to support efficient growth and energy provision. However, the rising cost of lipid sources continues to pose economic challenges to feed formulation and overall aquaculture profitability.

Atlantic salmon reared in freshwater environments exhibit a lower physiological sensitivity to dietary fatty acids, which has been interpreted as an evolutionary adaptation to natural dietary conditions [23]. In particular, freshwater-adapted Atlantic salmon and sockeye salmon have demonstrated greater capacities for fatty acid deposition in muscle tissues and more efficient lipid biosynthesis, which are associated with improved growth rates and enhanced feed conversion efficiency [24,25,26]. These traits indicate distinct advantages in terms of metabolic efficiency and cost-effectiveness under freshwater aquaculture systems. Indeed, our previous studies assessing the physiological responses of parr-stage Atlantic salmon prior to smoltification under varying dietary lipid levels reported no significant differences in growth performance, antioxidant enzyme activity, lipid metabolism, or expression of immune-related genes [27]. These findings suggest that early life stages of Atlantic salmon may not require high dietary lipid intake and that lipid levels have a limited physiological impact in the absence of seawater transfer. Consequently, low-lipid feed strategies may be viable in freshwater-based rearing systems. Recently, there has been growing interest in developing rearing technologies that enable the full life cycle of Atlantic salmon to be completed in freshwater environments [28,29]. Accordingly, the present study aims to evaluate the graded dietary lipid inclusion of Atlantic salmon reared entirely in freshwater, thereby providing foundational data to support the formulation of cost-effective and nutritionally optimized feeds for sustainable salmon aquaculture.

## 2. Materials and Methods

### 2.1. Experimental Diets

Four experimental diets were formulated to contain identical crude protein levels (45%) but varying lipid levels: 14.02% (L14), 16.51% (L16), 18.61% (L18), and 20.35% (L20). To compensate for variation in lipid inclusion across treatments, starch was used as a non-lipid substitute so that the total formulation remained constant. The formulation and proximate composition of diets are presented in Table 1. The proximate composition was determined in duplicate, and the mean values are reported. Fish oil and soybean oil were used as lipid sources and incorporated at a 1:1 ratio in all diets. In brief, feed ingredients and water were thoroughly mixed for 30 min using a vertical mixer (HYVM-1214; Hanyoung Corp., Hanam, Republic of Korea). The mixed feed was then pelleted using a pelletizer (SP-75; Geumgang ENG, Busan, Republic of Korea), followed by drying at 60 °C for 2 h in a hot air dryer (SHI-300; Shinhanil, Gwangju, Republic of Korea). The diets were stored at −20 °C until use.

### 2.2. Experimental Fish and Feeding Trial

Atlantic salmon used in this study were purchased as eggs from Benchmark Genetics (Hafnarfjörður, Iceland). Eggs hatched, and juvenile salmon were raised at the Gangwon-do Inland Fisheries Resource Center. All fish were raised in freshwater without undergoing transfer to seawater and were fully acclimated to the freshwater environment. The initial weight and total length of the fish used in the experiment were 241.5 ± 9.7 g and 30.6 ± 0.2 cm, respectively. A total of 200 fish were randomly distributed into four 1000-L circular fiberglass tanks (50 fish per tank) equipped with a recirculating aquaculture system and filtration unit. The fish were acclimated to experimental conditions in the same tanks for two weeks before the experiment began. A 12-week feeding trial was then conducted.

Experimental diets were provided twice daily to apparent satiation (09:00 and 16:00). During the feeding trial, pellet stability was visually monitored under freshwater conditions, and pellets remained intact until consumption, with no apparent disintegration observed. Water quality was monitored daily using a commercial water analysis kit (HUMAS, Daejeon, Republic of Korea). Throughout the trial, the water temperature was maintained at approximately 15 °C using a cooling and heating tank system, with pH 6.5–7.0, dissolved oxygen (DO) levels of 9–11 mg/L, and ammonia concentrations below 1 mg/L across all tanks.

### 2.3. Sampling

At the end of the 12-week feeding trial, sixteen fish per tank were randomly selected and anesthetized in ethyl 3-aminobenzoate methanesulfonate solution (100 mg/L; Sigma-Aldrich, St. Louis, MO, USA) for biometric measurements (total length and body weight). These measurements were used to evaluate growth performance, feed utilization, and condition indices

Among these, three individuals were immediately frozen at −20 °C for subsequent whole-body proximate composition analysis. Blood samples were collected from the caudal vasculature of the remaining four fish using sterile syringes. Blood was collected into 1.5 mL tubes coated with 20 μL of heparin (BD Microtainer, Franklin Lakes, NJ, USA) for each sample. Plasma was separated by centrifugation at 3000 rpm for 15 min at 4 °C and stored at −80 °C for further biochemical analyses. Biochemical parameters included antioxidant enzymes such as superoxide dismutase (SOD) and glutathione peroxidase (GPx), innate immune parameter lysozyme (LZM), and stress and health indicators such as glutamic-pyruvic transaminase (GPT), glutamic-oxaloacetic transaminase (GOT), glucose, and total protein (TP) concentrations.

Following blood collection, the viscera and liver were excised and weighed to determine viscerosomatic and hepatosomatic indices. A portion of the liver tissue was collected in 1.5 mL microcentrifuge tubes and immediately stored at −80 °C for subsequent molecular analyses, including RNA sequencing.

### 2.4. Proximate and Fatty Acid Composition of the Experimental Diets and Whole-Body

Experimental diets and whole-body samples from five randomly selected fish per tank were homogenized prior to analysis. Proximate composition was determined according to standard methods described by the AOAC. Moisture content was analyzed via oven-drying at atmospheric pressure (135 °C for 2 h). Crude protein was determined using the Kjeldahl method (nitrogen × 6.25) [30], and crude ash was determined using a muffle furnace. Moisture, ash and lipid contents were determined according to the methods described previously [31]. Crude lipid content was analyzed via Soxhlet extraction using samples that had been freeze-dried for 12 h (Soxtec system 1046; Tecator AB, Hilleroed, Sweden).

For the fatty acid analysis, total lipids from experimental diets and fish samples were extracted by following a previous method [32]. The extracted lipids were methylated using 14% boron trifluoride-methanol (Sigma-Aldrich, USA) to obtain fatty acid methyl esters. Fatty acid profiles were determined using gas chromatography (GC, Clarus 600; PerkinElmer, Waltham, MA, USA) equipped with a capillary column (SP™-2560, 100 m × 0.25 mm i.d., film thickness 0.20 μm; Supelco, Bellefonte, PA, USA). Helium was used as the carrier gas. The oven temperature was initially set at 140 °C and increased to 240 °C at a rate of 4 °C/min. Identification and quantification of fatty acids were performed by comparison with a known standard mixture (PUFA 37 Component FAME Mix; Supelco, Bellefonte, PA, USA). The detailed fatty acid composition of each experimental diet is presented in Table 2.

### 2.5. Hematobiochemical Analysis

Plasma samples from each treatment group were thawed completely on ice prior to analysis. Each sample was diluted 1:200 in the assay buffer prior to measurement. Antioxidant and immune-related parameters, including SOD, GPx, and LZM, were quantified using commercially available FISH-specific ELISA kits (CUSABIO, Houston, TX, USA), following the manufacturer’s instructions.

Clinical biochemical parameters, including GPT, GOT, glucose, and TP, were analyzed using commercial diagnostic kits (Asan Pharm, Seoul, Republic of Korea) according to the manufacturer’s protocols. Absorbance was measured using a microplate spectrophotometer (TECAN Infinite 200 Pro M, Männedorf, Switzerland) at wavelengths of 450 nm, 505 nm, 540 nm, and 570 nm, depending on the assay. Standard curves and sample concentrations were calculated using CurveExpert software (version 1.3).

### 2.6. RNA Sequencing and Transcriptomic Analysis

Liver tissue samples collected from the L14 and L16 dietary groups were used for transcriptomic analysis. For RNA-seq analysis, RNA was extracted from three fish per dietary treatment. Equal amounts of RNA from each individual were then pooled within each group prior to library preparation and sequencing. Total RNA was extracted using the QIAzol Lysis Reagent and RNeasy Mini Kit (Qiagen, Hilden, Germany), followed by DNase treatment to remove genomic DNA contamination. RNA libraries were prepared using the TruSeq Stranded Total RNA kit with Ribo-Zero (Illumina, San Diego, CA, USA). Purified RNA was randomly fragmented, and the resulting RNA fragments were reverse-transcribed into cDNA. The cDNA was then ligated with adapters, amplified by PCR, and size-selected to include insert sizes ranging from 200 to 400 bp.

Sequencing was performed using the NovaSeq 6000 platform (Illumina, San Diego, CA, USA) with paired-end reads (101 bp). Raw sequencing reads underwent quality control to remove low-quality reads, adapter sequences, contaminant DNA, and PCR duplicates. Clean reads were aligned to the reference genome (Ssal_v3.1) using HISAT2, and transcript assembly was conducted with StringTie (version 2.2.3). Transcript abundance was quantified using transcript per million of mapped reads (TPM). RNA-sequencing (RNA-seq) data processing and normalization were performed following methods described previously [33,34].

Differentially expressed genes (DEGs, an absolute fold change ≥ 2 and Benjamini-Hochberg adjusted *p* < 0.05) were identified based on statistical comparisons between dietary groups using hypothesis testing. Annotated DEGs were subjected to functional enrichment analysis using the Gene Ontology (GO) databases. GO enrichment analysis was conducted using gProfiler, covering the three GO domains: molecular function and cellular component.

### 2.7. Statistical Analysis

Fish growth performance parameters were expressed as the mean ± standard deviation (mean ± SD). Statistical analyses and data visualization were conducted using RStudio (version 2025.05.1+513), BioRender (BioRender.com) and CurveExpert (version 1.3). Normality and homogeneity of variance were assessed using the Shapiro–Wilk test and Bartlett’s test, respectively. One-way analysis of variance was then performed, and significant differences among groups were compared using Tukey’s honestly significant difference (*p* < 0.05). For non-parametric comparisons, the Kruskal–Wallis test was applied, followed by Dunn’s post hoc test to identify significant differences among experimental groups.

## 3. Result

### 3.1. Growth Performance and Feed Efficiency Ratio

Throughout the 12-week feeding trial, no pathological symptoms were observed in any experimental groups. Fish fed the L16 diet, containing 16.5% dietary lipid, exhibited the highest weight gain rate (WGR; 84.0 ± 9.1%) and feed efficiency ratio (FER; 102.7 ± 11.1%), representing the best growth performance among treatments. In contrast, fish fed the L14 diet (14% lipid) showed significantly lower WGR (44.9 ± 12.4%) and FER (72.3 ± 19.9%) than those fed the L16 diet (*p* = 0.049). Diets containing 18% and 20% lipids yielded comparable WGR values, with no significant differences between them (*p* = 0.991). Morphometric indices, including viscerosomatic index (VSI), hepatosomatic index (HSI), and condition factor (CF), did not differ significantly among dietary treatments (VSI: *p* = 0.965, HSI: *p* = 0.526, CF: *p* = 0.584) (Table 3).

### 3.2. Whole-Body Composition

#### 3.2.1. Proximate Composition

The proportion of whole-body protein decreased with increasing dietary lipid levels, with the highest value observed in fish fed the L14 diet (*p* < 0.001). Conversely, increasing dietary lipids resulted in higher whole-body lipid content, with the L20 group showing a significantly greater proportion than the other groups and L14 the lowest (*p* < 0.001). Whole-body ash content was highest in the L14 group and lowest in the L18 group (*p* = 0.004). Moisture content did not differ significantly among dietary treatments (*p =* 0.224) (Table 4).

#### 3.2.2. Fatty Acids Composition

Fish fed the L18 and L20 diets, which contained relatively higher dietary lipid levels, exhibited lower proportions of EPA and DHA in whole-body. In contrast, the lowest proportion of C15:0 was observed in fish fed the L14 diet, which contained the lowest dietary lipid level. The proportions of other fatty acids were comparable across all groups (Table 5).

### 3.3. Plasma Antioxidant Capacity and Biochemical Analysis

No significant differences in SOD activity were observed among dietary treatments (*p =* 0.553). However, GPx activity was highest in fish fed a diet containing 18% lipid and lowest in those fed the L14 diet (*p =* 0.041). LZM activity did not differ significantly among treatments (*p* = 0.797). Biochemical analyses revealed no significant differences in GPT, GOT, or TP values across dietary groups (GPT: *p =* 0.288, GOT: *p =* 0.798, TP: *p =* 0.211) (Table 6). Notably, plasma glucose concentration differed among dietary treatments (*p* = 0.017), with a significantly lower value in the L16 group compared with the L18 group (*p =* 0.020). No significant differences in glucose levels were detected among treatments other than L16.

### 3.4. Liver Transcriptomic Profiling Analysis

#### 3.4.1. Differentially Expressed Genes Analysis

**Figure 1:**
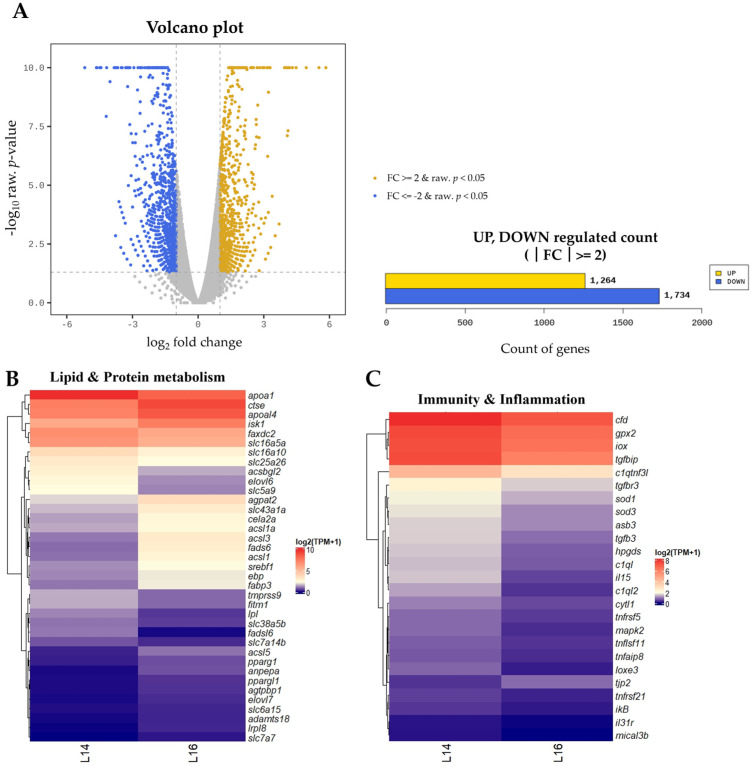
Transcriptomic Analysis of DEGs in L14 and L16 Diets. RNA was extracted from three fish per experimental group, equalized in quantity, pooled, and then used for RNA-seq analysis. (**A**) Volcano plot of DEGs in L14 and L16 experiments. Significantly upregulated or downregulated genes are indicated by yellow or blue dots (*p* < 0.05). Yellow dots indicate genes upregulated in L14 compared to L16, and blue dots indicate downregulated genes in L14 compared to L16. (**B**) Heatmap showing the expression levels of DEGs related to lipid and protein metabolism. (**C**) Heatmap showing the expression levels of DEGs related to immunity and inflammation. Gene expression levels were normalized and visualized as log_2_(TPM + 1) values. The color gradient, from navy to red, in the heatmap indicates increasing expression levels.

Based on the growth results, we selected the L14 and L16 treatments, which showed a significant difference in growth for RNA-seq. Comparative transcriptomic analysis between the L14 and L16 dietary groups identified a total of 2117 DEGs, comprising 905 upregulated and 1212 downregulated genes in the L14 group relative to the L16 group (Figure 1A and Supplementary Table S1). The overall expression patterns of DEGs are visualized using a heatmap. Genes (*apoal4*, *acsl1*, *acsl1a*, *acsl3*, *acsl5*, *ebp* and *lrpl8*) involved in fatty acid metabolism, lipogenesis and activation were significantly upregulated in L16 group. However, several proteases and amino acid transporter-related genes (*slc16a10*, *slc7a7*, *slc7a14b*, *slc25a26* and *slc38a5b*) displayed higher expression in the L14 group (Figure 1B). The expression levels of immune and inflammation-related genes were higher in the L14 dietary group than in the L16 group. Representative transcripts that were more highly expressed in the L14 group included antioxidant enzymes (*sod1*, *sod3* and *gpx2*), cytokine-related genes (*iox*, *tnfrsf21*, *tnflsf11*, *tnfaip8*, *tnfrsf5*, *il15* and *il31r*), regulators of cellular immunity and inflammation (*tgfb3*, *tgfbr3*, *tgfbip* and *asb3*) and complement-associated genes (*cfd*, *c1ql*, *c1ql2* and *c1qtnf3l*) (Figure 1C). 

#### 3.4.2. GO Functional Analysis

**Figure 2:**
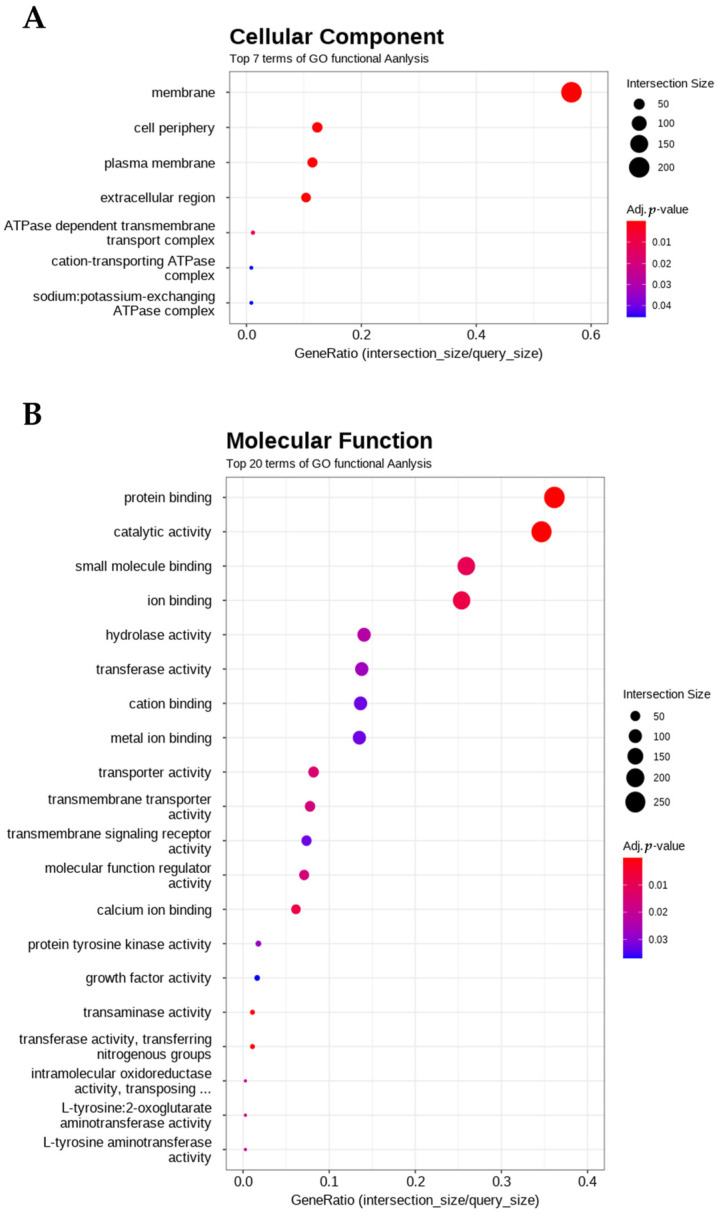
Functional analysis of GO terms. (**A**) GO terms for the top 7 cellular components among the entire DEG set. (**B**) GO terms for the top 20 molecular function among the entire DEG set. The size and color of the dots represent the number of DEG genes associated with the GO term and the adjusted *p*-value, respectively. Color intensity represents the statistical significance of enrichment (adjusted *p*-value), with red indicating more significant enrichment.

In the GO category of cellular components, the most significantly enriched terms included membrane, cell periphery, plasma membrane, extracellular region, ATPase-dependent transmembrane transport, cation-transporting ATPase, and sodium–potassium-exchanging ATPase. The DEGs associated with these terms indicated that the membrane was the most prominent GO term differentiating the L14 and L16 dietary groups (Figure 2A).

In the molecular function category, the top enriched terms were protein binding, catalytic activity, small molecule binding, and ion binding, along with specific terms such as transferase activity, transporter activity, transmembrane transporter activity and transmembrane signaling (Figure 2B).

## 4. Discussion

Atlantic salmon is one of the most economically important species in global aquaculture, and extensive nutritional research has focused on ensuring stable and sustainable production [35]. With the expansion of aquaculture, high-lipid diets have increasingly been used to promote rapid growth; however, excessive lipid intake has been associated with adverse physiological effects [36,37]. Atlantic salmon undergo smoltification and seawater migration, during which adequate essential fatty acids are critical for maintaining metabolic and osmoregulatory balance [38]. In contrast, juveniles reared in freshwater exhibit greater lipid biosynthetic capacity and a reduced dependence on dietary fatty acids [23]. Previous observations suggest that high-lipid diets are not essential for freshwater-reared Atlantic salmon, and that low-lipid feeding strategies may improve FER while supporting more efficient feed utilization [27]. Therefore, this study evaluated the effects of graded dietary lipid levels on growth performance, physiological responses, and transcriptomic profiles in Atlantic salmon reared entirely in freshwater. These findings provide fundamental insights for optimizing dietary lipid requirements in freshwater-based salmon aquaculture.

### 4.1. Inadequate Dietary Lipid Levels Impair the Growth Performance of Atlantic Salmon

WGR and SGR are key performance indices in aquaculture [39]. In the present study, fish fed the diet containing 16% lipid exhibited the highest WGR and SGR, whereas those fed the 14% lipid diet showed the lowest growth performance. In the study on rainbow trout [40], fish fed a low-lipid diet exhibited reduced WGR, which was attributed to insufficient supply of essential fatty acids. Similarly, it has been reported that in Atlantic salmon at the parr stage, growth rate did not increase proportionally with higher dietary lipid levels, and no significant differences were observed between diets containing 16% and 28% lipid [41]. This outcome was explained by delayed digestion and reduced absorption efficiency associated with excessive lipid intake. Although excessive lipid intake is frequently associated with somatic lipid deposition and increased VSI and HSI in fish [42,43,44,45,46], we detected no significant differences, indicating that dietary lipid levels of up to 20% did not promote visceral fat accumulation under the present experimental conditions. Collectively, these results indicate that dietary lipid levels of 16% or higher are adequate for freshwater-reared Atlantic salmon, whereas lower levels (≤14%) are inadequate and compromise growth performance.

### 4.2. An Appropriate Level of Dietary Lipid Intake Is Required to Maintain Efficient Body Lipid Composition

Whole-body composition is closely coupled to growth in fish [47]. Consistent with this, numerous studies have documented a significant positive correlation between dietary lipid level and whole-body lipid content [18,48,49,50], a pattern also observed in the present work. As whole-body lipid increased, the proportional contribution of protein declined. Similar inverse shifts between body lipid and protein have been reported in Atlantic salmon and marble goby (*Oxyeleotris marmorata*) with increasing lipid intake [48,51]. The optimal body composition for growth differs among fish species. Previous studies have reported that lipid levels are not necessarily proportional to growth performance [52,53]. The present results demonstrate that, under the current experimental conditions, a body composition containing approximately 57% protein and 35% lipid is most favorable for growth performance.

With respect to fatty acid profiles, fish fed comparatively lower dietary lipids exhibited higher whole-body EPA+DHA proportions. Analogous responses have been described in gilthead seabream (*Sparus aurata*), where the lowest dietary EPA+DHA supply yielded the highest DHA retention [54]. In European seabass (*Dicentrarchus labrax*), DHA deficiency elicited selective conservation and endogenous synthesis to compensate for limited supply [55], and other studies likewise report maintenance of tissue EPA+DHA despite reduced intake [56]. Notably, increased dietary fatty acid intake does not necessarily lead to proportional increases in whole-body EPA and DHA levels, as metabolomic evidence indicates that inadequate EPA and DHA may disrupt hepatic metabolic regulation and promote hepatic lipid accumulation [57]. In Atlantic salmon, limited essential fatty acid intake has been shown to trigger substantial endogenous production to offset dietary shortfalls [58,59]. Collectively, these observations suggest that the higher EPA+DHA proportions observed in fish fed lower dietary lipid levels may reflect selective retention and metabolic regulation of essential fatty acids rather than a direct linear response to dietary supply.

### 4.3. Appropriate Dietary Lipid Intake Is Essential for Maintaining the Health of Atlantic Salmon

SOD and GPx constitute first-line enzymatic defenses against reactive oxygen species (ROS), limiting lipid peroxidation and maintaining redox homeostasis in fish [60,61]. High-lipid feeding has been reported to disrupt lipid metabolism, exacerbate oxidative stress, and depress antioxidant enzyme activities in zebrafish (*Danio rerio*) [36] and carp (*Cyprinus carpio*) [62]. In contrast to these patterns, we detected no statistically significant differences in SOD activity among dietary treatments in the present study, whereas GPx activity was lowest at 14% dietary lipid and highest at 18%. The direction of this response is consistent with the elevated lipid requirements typical of cold-water salmonids, including Atlantic salmon and Amur grayling (*Thymallus arcticus grubei*) [13,63]. Although high EPA+DHA intake has been proposed to foster an oxidative milieu [64], variation in experimental diets did not alter the antioxidant status of Atlantic salmon under our conditions, suggesting that relatively high EPA+DHA did not precipitate measurable oxidative stress and that this species maintains robust redox control. Plasma glucose is a recognized proxy of metabolic stress and health in fish [65,66], and typically rises with dietary stress, inflammation, or metabolic dysregulation [67,68]. Notably, the lowest plasma glucose concentration occurred at 16% dietary lipid, indicating attenuated stress at this level. Taken together, these findings indicate that a dietary lipid level around 16% supports antioxidant capacity and physiological stability in freshwater-reared Atlantic salmon.

### 4.4. Low Dietary Lipid Intake Influences Lipid Metabolism and Affects the Structural Integrity of Cell Membranes

The liver is a principal organ for lipid metabolism and immune regulation [69,70]. To resolve treatment-specific hepatic responses, we performed RNA-seq on livers from fish fed the 14% and 16% lipid diets, which differed in growth performance. APOA4 (apolipoprotein A-IV), predominantly synthesized in the liver and functionally engaged with chylomicrons and high-density lipoproteins, facilitates intestinal lipid absorption and contributes to high-density lipoprotein remodeling [71]. Concordant with prior observations in fish, higher dietary lipids have been associated with *apoa4* upregulation, and a similar pattern was observed in the present study. ACSLs (acyl-CoA synthetase long-chain) activate fatty acids to acyl-CoA, thereby gating their entry into β-oxidation and lipid biosynthetic pathways and accelerating cellular fatty acid handling [72]. In our dataset, *acsl1*, *acsl1a*, *acsl3* and *acsl5* were upregulated in L16 relative to L14, consistent with augmented hepatic lipid uptake, activation, and downstream metabolism at 16% dietary lipid. Cholesterol regulates membrane fluidity and is essential for maintaining membrane structure and intracellular signal transduction [73,74,75]. In the present study, *ebp* and *lrp8*, which were upregulated in the L16 group, were identified as key regulators involved in cholesterol biosynthesis and lipid metabolism, respectively [74,76]. Consistent with this, a study demonstrated a positive correlation between LRP8 expression and the expression of lipid metabolism–related genes [77]. These genes are all associated with the cell membrane, and the enriched GO terms identified in this study included ‘membrane’, ‘cell periphery’ and ‘plasma membrane’ under the “cellular component” category. Together, these results indicate that dietary lipid levels are associated with differences in hepatic lipid metabolism–related gene expression, with the 16% lipid diet showing a transcriptomic profile more consistent with enhanced fatty acid synthesis and metabolism compared with 14%.

Members of the solute carrier family mediate amino acid and nutrient transport and are central to growth and metabolic homeostasis; their expression is broadly tuned by nutrient-sensing pathways [78,79]. Notably, amino-acid transporter genes (*slc16a10*, *slc7a7*, *slc7a14b*, *slc25a26*, *slc38a5b*) were comparatively higher in L14. Other studies interpret increased amino-acid transport capacity as a compensatory response to nutrient insufficiency [80,81]. The enriched GO terms under the “molecular function” category included ‘transferase activity’, ‘transporter activity’ and ‘transmembrane signaling’. Alterations in genes associated with transporter proteins may reflect impaired nutrient transport capacity. These results appear to be related to dietary nutrient composition, suggesting that the 14% lipid diet is associated with a transcriptional profile indicative of altered nutrient transport and metabolic regulation.

### 4.5. A Dietary Lipid Level Below the Optimal Range May Induce Nutritional Stress in Freshwater-Reared Atlantic Salmon

The complement system is tightly linked to inflammation and innate immunity and is frequently used as a marker of inflammatory activation [82,83]. Concordantly, several complement-associated genes (*c1ql2*, *c1ql*, *c1qtnf3l* and *cfd*) were upregulated in the L14 group, reflecting higher expression of inflammation-related genes compared with L16. Among cytokine pathways, IL-15, a member of the IL-2 family, induces IFN-γ and primarily signals through downstream cascades to modulate immune cell function [84]. In gilthead sea bream (*Sparus aurata*), increased *il15* expression has been attributed to dietary immunostimulants and associated cytokine responses [85], and in orange spotted grouper (*Epinephelus coioides*), expression levels of *il15* rises significantly following LPS or poly I:C exposure [86]. IL-31R, which binds IL-31, is typically elevated in mucosal and chronic inflammatory settings [87,88]. TNF, a potent pro-inflammatory cytokine, is well known to cause tissue injury when excessive [89] and can be induced not only by pathogens but also by thermal, salinity, and nutritional stress [90,91,92]. TGF-β is a pleiotropic cytokine broadly involved in immune regulation, and increased expression has been reported with soybean-meal enteritis, oxidative stress, and immune stimulation (LPS, poly I:C) [93,94]. Although TGF-β is commonly stored in a latent form, ROS can activate it by oxidatively modifying the latency-associated peptide, thereby altering its conformation and releasing active TGF-β [94]. Overall, the DEG analysis between the L14 and L16 groups showed higher expression of immune and inflammation-related genes in L14. This transcriptional pattern is consistent with the 14% lipid diet being associated with increased expression of genes related to immune and inflammatory pathways, which may reflect a state of nutritional stress.

## 5. Conclusions

This study evaluated the effects of dietary lipid levels in freshwater-reared Atlantic salmon. Across a 12-week feeding trial, diets containing ≥16.51% lipid produced superior growth performance, and accompanying whole-body composition patterns were consistent with these outcomes. Hepatic transcriptomic analyses further indicated that a 14% lipid regimen was associated with less favorable expression of genes involved in fatty acid metabolism and immune function, together with differences across membrane-associated processes, ion-related functions, and growth factor activity. Taken together, the results suggest that under the present freshwater rearing conditions, a dietary lipid level of approximately 16% appears to be a practical and suitable target within the range tested for guiding lipid inclusion. These findings provide a foundation for the future development of feeds for freshwater-reared Atlantic salmon. However, because this study was conducted at a single life stage over a 12-week period, lipid requirements may differ during subsequent growth phases or during smoltification; therefore, longer-term studies spanning later growth stages are warranted to confirm the durability of the observed effects. In addition, in future studies, we will use the key genes identified in this RNA-seq analysis as targets for qPCR to further refine and validate the molecular responses to dietary lipid levels.

## Figures and Tables

**Table 1:** Formulation of ingredients and proximate composition of experimental diets (%, dry matter basis).

Ingredients (%)	Experimental Diet			
	L14	L16	L18	L20
Fish meal ^1^	30.00	30.00	30.00	30.00
Soybean meal ^2^	8.00	8.00	8.00	8.00
SPC ^3^	19.00	19.00	19.00	19.00
Chicken meal ^4^	4.00	4.00	4.00	4.00
Wheat gluten ^5^	10.00	10.00	10.00	10.00
Wheat flour ^6^	3.80	3.80	3.80	3.80
α-starch ^7^	10.00	8.00	6.00	4.00
Fish oil ^8^	5.60	6.60	7.60	8.60
Soybean oil ^9^	5.60	6.60	7.60	8.60
Vitamin premix ^10^	2.00	2.00	2.00	2.00
Mineral premix ^11^	2.00	2.00	2.00	2.00
Proximate composition (%)				
Moisture	5.09	4.50	4.35	4.38
Crude protein	47.96	47.63	47.09	47.24
Crude lipid	14.02	16.51	18.61	20.35
Ash	8.98	8.97	9.06	9.28

^1^ BLUMAR Co., Ltd., Las Condes, Santiago, Chile (Fish meal, crude protein: 67.61% and crude lipid: 7.49).^2^ The Feed Co., Ltd., Seoul, Republic of Korea (Soybean meal, crude protein: 43% and crude lipid: 2%). ^3^ The Feed Co., Ltd., Seoul, Republic of Korea (SPC, soy protein concentrate, crude protein: 61% and crude lipid: 0.2%). ^4^ The Feed Co., Ltd., Seoul, Republic of Korea (Chicken meal derived from chicken slaughter, crude protein: 32.5% and crude lipid: 0.27%). ^5^ Royal Lion Co., Ltd., Alkmaar, The Netherlands (Wheat gluten, crude protein: 77.62% and lipid: 1.3%). ^6^ The Feed Co., Ltd., Seoul, Republic of Korea (Wheat flour, crude protein: 14.31% and crude lipid: 2.26%). ^7^ The Feed Co., Ltd., Seoul, Republic of Korea (α-starch, crude protein: 0.13% and crude lipid: 0.01%). ^8^ The Feed Co., Ltd., Seoul, Republic of Korea (Pollock oil). ^9^ Nabiton Co., Ltd., Hampyeong, Republic of Korea (Soybean oil). ^10^ FeedBest Co., Ltd., Cheonan, Republic of Korea (Vitamin premix: vitamin A, 5000 IU/g; vitamin D3, 1000 IU/g; vitamin E, 38 mg/g). ^11^ FeedBest Co., Ltd., Cheonan, Republic of Korea (Mineral premix: zinc, 14 mg/kg; copper, 2 mg/kg; iron, 13 mg/kg; manganese, 12 mg/kg).

**Table 2:** Fatty acid compositions of experimental diets (% of total fatty acids).

Fatty Acids	Experimental Diets			
	L14	L16	L18	L20
C14:0 ^1^	9.57	9.28	9.34	9.32
C15:0 ^2^	0.31	0.29	0.30	0.28
C16:0 ^3^	31.44	31.25	31.10	30.45
C16:1 ^4^	2.82	2.77	2.76	2.70
C18:0 ^5^	7.17	7.29	7.17	7.15
C18:1n9c ^6^	10.95	11.33	11.17	11.28
C18:2n6c ^7^	22.09	22.66	22.95	23.43
C20:0 ^8^	0.51	0.53	0.52	0.53
C20:1 ^9^	1.44	1.56	1.55	1.53
C18:3n3 ^10^	2.83	2.82	2.88	2.95
C20:2 ^11^	0.10	0.09	0.06	0.09
C22:0 ^12^	0.38	0.39	0.38	0.41
C22:1n9 ^13^	0.33	0.31	0.35	0.34
C24:0 ^14^	0.13	0.14	0.14	0.13
C20:5n3 ^15^	5.82	5.42	5.49	5.58
C24:1 ^16^	0.15	0.16	0.17	0.16
C22:6n3 ^17^	3.96	3.71	3.67	3.67
Total	100	100	100	100
n-3	12.61	11.95	12.04	12.20
n-6	22.09	22.66	22.95	23.43
n-3/n-6	0.57	0.53	0.52	0.52

^1^ C14:0, Myristic acid; ^2^ C15:0, Pentadecanoic acid; ^3^ C16:0, Palmitic acid; ^4^ C16:1, Palmitoleic acid; ^5^ C18:0, Stearic acid; ^6^ C18:1n9c, Oleic acid; ^7^ C18:2n6c, Linoleic acid; ^8^ C20:0, Arachidic acid; ^9^ C20:1, cis-11-Eicosenoic acid; ^10^ C18:3n3, Linolenic acid; ^11^ C20:2, Eicosadienoic acid; ^12^ C22:0, Behenic acid; ^13^ C22:1n9, Erucic acid; ^14^ C24:0, Lignoceric acid; ^15^ C20:5n3, Eicosapentaenoic acid; ^16^ C24:1, Nervonic acid; ^17^ C22:6n3, Docosahexaenoic acid.

**Table 3:** Growth performance, feed efficiency ratio and morphometric parameters of fish fed the four experimental diets for 12 weeks.

Parameters (g, %)	Experimental Diets				
	L14	L16	L18	L20	*p*-Value
WGR ^1^	44.9 ± 49.5 ^b^	84.0 ± 36.4 ^a^	75.4 ± 46.2 ^ab^	75.7 ± 31.1 ^ab^	0.049
SGR ^2^	0.38 ± 0.42 ^b^	0.71 ± 0.25 ^a^	0.63 ± 0.38 ^ab^	0.66 ± 0.23 ^ab^	0.030
FER ^3^	72.3 ± 19.9	102.7 ± 11.1	89.3 ± 13.7	95.5 ± 9.8	0.479
CF ^4^	0.90 ± 0.21	1.01 ± 0.13	0.94 ± 0.25	0.98 ± 0.15	0.584
VSI ^5^	8.2 ± 0.7	7.9 ± 1.0	8.3 ± 0.9	8.2 ± 2.1	0.965
HSI ^6^	1.1 ± 0.1	1.2 ± 0.1	1.1 ± 0.1	1.3 ± 0.3	0.526

Values are presented as mean ± SD. ^1^ WGR, weight gain rate = 100 × (final mean body weight − initial mean body weight)/initial mean body weight. ^2^ SGR, specific growth rate = 100 × (ln final weight − ln initial weight)/days. ^3^ Feed efficiency ratio = 100 × (wet weight gain/dry feed intake). ^4^ CF, condition factor = 100 × (fish weight/total body length^3^). ^5^ VSI, viscerosomatic index = 100 × (viscera weight/whole body weight). ^6^ HSI, hepatosomatic index = 100 × (liver weight/whole body weight). Values in the same row with different superscript letters are significantly different (*p* < 0.05). WGR, SGR, FER, and CF, *n* = 16; VSI and HSI, *n* = 5.

**Table 4:** Proximate composition of fish whole-body.

Parameters (%)	Experimental Diets				
	L14	L16	L18	L20	*p*-Value
Protein	59.18 ± 0.47 ^a^	57.38 ± 0.22 ^b^	55.76 ± 0.31 ^c^	53.22 ± 0.04 ^d^	<0.001
Lipid	32.58 ± 0.08 ^d^	35.10 ± 0.29 ^c^	37.62 ± 0.07 ^b^	39.90 ± 0.12 ^a^	<0.001
Ash	8.12 ± 0.06 ^a^	7.45 ± 0.04 ^b^	6.63 ± 0.03 ^c^	6.88 ± 0.02 ^bc^	<0.001
Moisture	68.78 ± 0.20	69.45 ± 0.15	69.3 ± 0.18	68.96 ± 0.31	0.224

Values are presented as mean ± SD from duplicate measurements of pooled samples obtained from three fish. Protein, lipid, and ash contents were expressed on a dry matter basis, whereas moisture content was expressed on a wet weight basis. Values in the same row with different superscript letters are significantly different (*p* < 0.05).

**Table 5:** Fatty acid compositions of fish whole-body (% of total fatty acid).

Fatty Acids (%)	Experimental Diets			
	L14	L16	L18	L20
C14:0 ^1^	6.95	6.96	7.49	7.4
C15:0 ^2^	0.13	0.35	0.36	0.36
C16:0 ^3^	34.16	34.25	33.73	33.72
C16:1 ^4^	2.48	2.45	2.49	2.52
C18:0 ^5^	10.17	10.13	9.87	9.93
C18:1n9c ^6^	14.87	14.94	15.02	14.93
C18:2n6c ^7^	15.78	15.65	17.54	17.41
C20:0 ^8^	0.41	0.4	0.43	0.43
C18:3n6 ^9^	0.28	0.31	0.26	0.29
C20:1 ^10^	1.67	1.63	1.7	1.79
C18:3n3 ^11^	1.76	1.68	1.78	1.81
C20:2 ^12^	1.29	1.2	1.34	1.38
C22:0 ^13^	0.22	0.22	0.24	0.25
C20:3n6 ^14^	0.56	0.56	0.47	0.46
C22:1n9 ^15^	0.23	0.24	0.27	0.24
C20:3n3 ^16^	0.16	0.15	0	0
C20:4n6 ^17^	0.36	0.36	0.27	0.26
C20:5n3 ^18^	1.72	1.74	1.48	1.47
C24:1 ^19^	0.17	0.19	0.19	0.2
C22:6n3 ^20^	6.63	6.59	5.07	5.15
Total	100	100	100	100
n-3	10.11	10.01	8.33	8.43
n-6	16.98	16.88	18.54	18.42
n-3/n-6	0.60	0.59	0.45	0.46
EPA+DHA	8.35	8.33	6.55	6.62
DHA:EPA	0.26	0.26	0.29	0.29

Values represent the mean of two measurements obtained from pooled samples (*n* = 3). ^1^ C14:0, Myristic acid; ^2^ C15:0, Pentadecanoic acid; ^3^ C16:0, Palmitic acid; ^4^ C16:1, Palmitoleic acid; ^5^ C18:0, Stearic acid; ^6^ C18:1n9c, Oleic acid; ^7^ C18:2n6c, Linoleic acid; ^8^ C20:0, Arachidic acid; ^9^ C18:3n6, Gamma linolenic acid; ^10^ C20:1, cis-11-Eicosenoic acid; ^11^ C18:3n3, Linolenic acid; ^12^ C20:2, Eicosadienoic acid; ^13^ C22:0, Behenic acid; ^14^ C20:3n6, Dihomo-γ-linolenic acid; ^15^ C22:1n9, Erucic acid; ^16^ C20:3n3, Eicosatrienoic acid; ^17^ C20:4n6, Arachidonic acid; ^18^ C20:5n3, Eicosapentaenoic acid; ^19^ C24:1, Nervonic acid; ^20^ C22:6n3, Docosahexaenoic acid.

**Table 6:** Antioxidant and biochemical parameters in fish fed experimental diets.

Parameters	Experimental Diets				
	L14	L16	L18	L20	*p*-Value
SOD (ng/mL)	3600 ± 1237	2947 ± 420	3246 ± 668	3894 ± 1210	0.553
GPx (U/mL)	714 ± 58 ^b^	859 ± 93 ^ab^	892 ± 22 ^a^	751 ± 118 ^ab^	0.027
LZM (ng/mL)	556 ± 330	611 ± 408	534 ± 315	424 ± 156	0.797
GPT (U/mL)	29.1 ± 3.1	26.6 ± 3.5	30.1 ± 0.6	29.3 ± 1.8	0.288
GOT (U/mL)	143.5 ± 8.3	144.6 ± 12.4	151.5 ± 14.9	145.1 ± 13.0	0.798
Glucose (mg/dL)	60.71 ± 0.86 ^ab^	58.24 ± 1.23 ^b^	67.30 ± 2.87 ^a^	61.61 ± 9.33 ^ab^	0.017
TP (g/dL)	7.91 ± 0.05	8.49 ± 0.90	7.81 ± 1.30	6.61 ± 1.61	0.211

Values are presented as mean ± SD (*n* = 4). Values in the same row with different superscript letters are significantly different (*p* < 0.05).

## Data Availability

The data supporting the findings of this study are included within the article and its Supplementary Materials.

## Supplementary Materials

The following supporting information can be downloaded at https://www.mdpi.com/article/10.3390/ani16030356/s1. Table S1: List of differentially expressed genes between L14 and L16 groups.

## References

[1] Wilfart A., Garcia-Launay F., Terrier F., Soudé E., Aguirre P., Skiba-Cassy S. (2023). A step towards sustainable aquaculture: Multiobjective feed formulation reduces environmental impacts at feed and farm levels for rainbow trout. Aquaculture.

[2] Torres-Maravilla E., Parra M., Maisey K., Vargas R.A., Cabezas-Cruz A., Gonzalez A., Tello M., Bermúdez-Humarán L.G. (2024). Importance of probiotics in fish aquaculture: Towards the identification and design of novel probiotics. Microorganisms.

[3] Nederlof M.A., Verdegem M.C., Smaal A.C., Jansen H.M. (2022). Nutrient retention efficiencies in integrated multi-trophic aquaculture. Rev. Aquac..

[4] Kong W., Huang S., Yang Z., Shi F., Feng Y., Khatoon Z. (2020). Fish Feed Quality Is a Key Factor in Impacting Aquaculture Water Environment: Evidence from Incubator Experiments. Sci. Rep..

[5] Davis D.A., Hardy R.W. (2022). Feeding and fish husbandry. Fish Nutrition.

[6] Hardy R.W., Kaushik S.J., Mai K., Bai S.C. (2022). Fish nutrition—History and perspectives. Fish Nutrition.

[7] Zhang G., Guan J., Chen F., Xu J., Su N., Zhang H., Wang Z., Wang S., Xu C., Xie D. (2025). Effects of dietary lipid source and level on growth performance, antioxidant capacity, and hepatic metabolism in marine teleost *Trachinotus ovatus*: Insights from fatty acid composition. Aquac. Rep..

[8] Fang H.-H., Zhao W., Xie J.-J., Yin P., Zhuang Z.-X., Liu Y.-J., Tian L.-X., Niu J. (2021). Effects of dietary lipid levels on growth performance, hepatic health, lipid metabolism and intestinal microbiota on *Trachinotus ovatus*. Aquac. Nutr..

[9] Liang P., Wang Y., Wu L., Qin Z., Lai M., Lin J., Shao J., Zhang D. (2025). Optimal dietary lipid requirement of *Spinibarbus caldwelli*: Comprehensive characterization of growth performance, feed utilization, serum biochemical and immune parameters, hepatic lipid metabolism and health maintenance. Aquaculture.

[10] Ren Y., Wei S., Yu H., Xing W., Xu G., Li T., Luo L. (2021). Dietary lipid levels affect growth, feed utilization, lipid deposition, health status and digestive enzyme activities of juvenile Siberian sturgeon, *Acipenser baerii*. Aquac. Nutr..

[11] Natnan M.E., Low C.F., Chong C.M., Daud N.I.N.A.A., Om A.D., Baharum S.N. (2022). Comparison of different dietary fatty acids supplement on the immune response of hybrid grouper (*Epinephelus fuscoguttatus* × *Epinephelus lanceolatus*) challenged with Vibrio vulnificus. Biology.

[12] Carr I., Glencross B., Santigosa E. (2023). The importance of essential fatty acids and their ratios in aquafeeds to enhance salmonid production, welfare, and human health. Front. Anim. Sci..

[13] Fan Z., Li J., Zhang Y., Wu D., Zheng X., Wang C., Wang L. (2021). Excessive dietary lipid affecting growth performance, feed utilization, lipid deposition, and hepatopancreas lipometabolism of large-sized common carp (*Cyprinus carpio*). Front. Nutr..

[14] Liao Z., Sun B., Zhang Q., Jia L., Wei Y., Liang M., Xu H. (2020). Dietary bile acids regulate the hepatic lipid homeostasis in tiger puffer fed normal or high-lipid diets. Aquaculture.

[15] Naiel M.A., Negm S.S., Ghazanfar S., Shukry M., Abdelnour S.A. (2023). The risk assessment of high-fat diet in farmed fish and its mitigation approaches: A review. J. Anim. Physiol. Anim. Nutr..

[16] Suo X., Yan X., Tan B., Pan S., Li T., Liu H., Huang W., Zhang S., Yang Y., Dong X. (2022). Lipid metabolism disorders of hybrid grouper (♀ *Epinephelus fuscointestinestatus* × ♂ *E. lanceolatu*) induced by high-lipid diet. Front. Mar. Sci..

[17] Stefansson S.O., Björnsson B.T., Ebbesson L.O., McCormick S.D. (2020). Smoltification. Fish Larval Physiology.

[18] Mota V.C., Verstege G.C., Striberny A., Lutfi E., Dessen J.-E., Sveen L., Burgerhout E., Bou M. (2024). Smoltification, seawater performance, and maturation in Atlantic salmon (*Salmo salar*) fed different fat levels. Front. Aquac..

[19] Ahmed I., Jan K., Fatma S., Dawood M.A. (2022). Muscle proximate composition of various food fish species and their nutritional significance: A review. J. Anim. Physiol. Anim. Nutr..

[20] Tocher D.R. (2003). Metabolism and Functions of Lipids and Fatty Acids in Teleost Fish. Rev. Fish. Sci..

[21] Samways K.M., Blair T.J., Charest M.A., Cunjak R.A. (2017). Effects of spawning Atlantic salmon (*Salmo salar*) on total lipid content and fatty acid composition of river food webs. Ecosphere.

[22] Rosenlund G., Torstensen B.E., Stubhaug I., Usman N., Sissener N.H. (2016). Atlantic salmon require long-chain n-3 fatty acids for optimal growth throughout the seawater period. J. Nutr. Sci..

[23] Betancor M., Olsen R.E., Solstorm D., Skulstad O.F., Tocher D.R. (2016). Assessment of a land-locked Atlantic salmon (*Salmo salar* L.) population as a potential genetic resource with a focus on long-chain polyunsaturated fatty acid biosynthesis. Biochim. Biophys. Acta (BBA)-Mol. Cell Biol. Lipids.

[24] Colombo S.M., Emam M., Peterson B.C., Hall J.R., Burr G., Zhang Z., Rise M.L. (2021). Freshwater, landlocked grand lake strain of atlantic salmon (*Salmo salar* L.) as a potential genetic source of long chain polyunsaturated fatty acids synthesis. Front. Mar. Sci..

[25] Rollin X., Peng J., Pham D., Ackman R.G., Larondelle Y. (2003). The effects of dietary lipid and strain difference on polyunsaturated fatty acid composition and conversion in anadromous and landlocked salmon (*Salmo salar* L.) parr. Comp. Biochem. Physiol. Part B Biochem. Mol. Biol..

[26] Gladyshev M.I., Lepskaya E.V., Sushchik N.N., Makhutova O.N., Kalachova G.S., Malyshevskaya K.K., Markevich G.N. (2012). Comparison of Polyunsaturated Fatty Acids Content in Filets of Anadromous and Landlocked Sockeye Salmon *Oncorhynchus Nerka*. J. Food Sci..

[27] Lee B., Lee J., Lim S., Seong M., Yun H., Han S., Kim K.-W., Lee S., Jeong S.-M., Park M.C. (2024). Effects of Low-Lipid Diets on Growth, Haematology, Histology and Immune Responses of Parr-Stage Atlantic Salmon (*Salmo salar*). Animals.

[28] Crouse C., Davidson J., May T., Summerfelt S., Good C. (2021). Production of market-size European strain Atlantic salmon (*Salmo salar*) in land-based freshwater closed containment aquaculture systems. Aquac. Eng..

[29] Gonzalez C., Gallardo-Hidalgo J., Yáñez J.M. (2022). Genotype-by-environment interaction for growth in seawater and freshwater in Atlantic salmon (*Salmo salar*). Aquaculture.

[30] Kjeldahl C. (1883). A new method for the determination of nitrogen in organic matter. Z. Anal. Chem..

[31] Park Y., Park M., Hamidoghli A., Kim C.-H., Bai S.C. (2021). Optimum dietary processed sulfur (Immuno-F) level has antibiotic effects on the growth, hematology and disease resistance of juvenile olive flounder, *Paralichthys olivaceus*. Anim. Feed. Sci. Technol..

[32] Metcalfe L.D., Schmitz A.A. (1961). The Rapid Preparation of Fatty Acid Esters for Gas Chromatographic Analysis. Anal. Chem..

[33] Park Y., Zhang Q., Fernandes J.M., Wiegertjes G.F., Kiron V. (2021). Macrophage heterogeneity in the intestinal cells of salmon: Hints from transcriptomic and imaging data. Front. Immunol..

[34] Park Y., Zhang Q., Wiegertjes G.F., Fernandes J.M., Kiron V. (2020). Adherent intestinal cells from Atlantic salmon show phagocytic ability and express macrophage-specific genes. Front. Cell Dev. Biol..

[35] Albrektsen S., Kortet R., Skov P.V., Ytteborg E., Gitlesen S., Kleinegris D., Mydland L.T., Hansen J.Ø., Lock E.J., Mørkøre T. (2022). Future feed resources in sustainable salmonid production: A review. Rev. Aquac..

[36] Jafari S., Akhavan-Bahabadi M., Shekarabi S.P.H., Mehrgan M.S., Lowen E., Lotfi K., Azari A. (2025). Astaxanthin-rich Nannochloropsis oculata mitigates high-fat diet-induced liver dysfunction in zebrafish by modulating lipid metabolism and oxidative stress. Sci. Rep..

[37] Sarker P.K. (2023). Microorganisms in fish feeds, technological innovations, and key strategies for sustainable aquaculture. Microorganisms.

[38] Codabaccus M.B., Bridle A.R., Nichols P.D., Carter C.G. (2011). Effect of feeding Atlantic salmon (*Salmo salar* L.) a diet enriched with stearidonic acid from parr to smolt on growth and n-3 long-chain PUFA biosynthesis. Br. J. Nutr..

[39] Lugert V., Thaller G., Tetens J., Schulz C., Krieter J. (2016). A review on fish growth calculation: Multiple functions in fish production and their specific application. Rev. Aquac..

[40] Chen Y.-F., Cao K.-L., Huang H.-F., Li X.-Q., Leng X.-J. (2023). Dietary effects of lipid and protein levels on growth, feed utilization, lipid metabolism, and antioxidant capacity of triploid rainbow trout (*Oncorhynchus mykiss*). Aquac. Nutr..

[41] Siciliani D., Kortner T.M., Berge G.M., Hansen A.K., Krogdahl Å. (2023). Effects of dietary lipid level and environmental temperature on lipid metabolism in the intestine and liver, and choline requirement in Atlantic salmon (*Salmo salar* L.) parr. J. Nutr. Sci..

[42] Guo D., Xie M., Xiao H., Xu L., Zhang S., Chen X., Wu Z. (2022). Bacillus subtilis supplementation in a high-fat diet modulates the gut microbiota and ameliorates hepatic lipid accumulation in grass carp (*Ctenopharyngodon idella*). Fishes.

[43] Wang J.-T., Liu Y.-J., Tian L.-X., Mai K.-S., Du Z.-Y., Wang Y., Yang H.-J. (2005). Effect of dietary lipid level on growth performance, lipid deposition, hepatic lipogenesis in juvenile cobia (*Rachycentron canadum*). Aquaculture.

[44] Wang J., Han T., Li X., Yang Y., Yang M., Hu S., Jiang Y., Harpaz S. (2017). Effects of dietary protein and lipid levels with different protein-to-energy ratios on growth performance, feed utilization and body composition of juvenile red-spotted grouper, *E pinephelus akaara*. Aquac. Nutr..

[45] Xie R.-T., Amenyogbe E., Chen G., Huang J.-S. (2021). Effects of feed fat level on growth performance, body composition and serum biochemical indices of hybrid grouper (*Epinephelus fuscoguttatus* × *Epinephelus polyphekadion*). Aquaculture.

[46] Chen Y., Lawson R., Shandilya U., Chiasson M.A., Karrow N.A., Huyben D. (2023). Dietary protein, lipid and insect meal on growth, plasma biochemistry and hepatic immune expression of lake whitefish (*Coregonus clupeaformis*). Fish Shellfish. Immunol. Rep..

[47] Cook J.T., McNiven M.A., Richardson G.F., Sutterlin A.M. (2000). Growth rate, body composition and feed digestibility/conversion of growth-enhanced transgenic Atlantic salmon (*Salmo salar*). Aquaculture.

[48] HEMRE, SANDNES (1999). Effect of dietary lipid level on muscle composition in Atlantic salmon *Salmo salar*. Aquac. Nutr..

[49] Berrill I.K., Porter M.J., Bromage N.R. (2004). The influence of dietary lipid inclusion and daily ration on growth and smoltification in 1+ Atlantic salmon (*Salmo salar*) parr. Aquaculture.

[50] Xie S., Lin Y., Wu T., Tian L., Liang J., Tan B. (2021). Dietary lipid levels affected growth performance, lipid accumulation, inflammatory response and apoptosis of Japanese seabass (*Lateolabrax japonicus*). Aquac. Nutr..

[51] Yong A.S.K., Ooi S., Shapawi R., Biswas A.K., Kenji T. (2015). Effects of dietary lipid increments on growth performance, feed utilization, carcass composition and intraperitoneal fat of marble goby, *Oxyeleotris marmorata*, juveniles. Turk. J. Fish. Aquat. Sci..

[52] Nguyen M.C., Fotedar R., Pham H.D. (2022). Effects of dietary protein and lipid levels on growth performance, feed utilization and body composition of juvenile giant trevally (*Caranx ignobilis* Forsskal, 1775). Aquac. Res..

[53] Wang J., Liu T., Zheng P., Xu H., Su H., Tao H., Yang Y. (2021). Effect of dietary lipid levels on growth performance, body composition, and feed utilization of juvenile spotted knifejaw *Oplegnathus punctatus*. Aquac. Rep..

[54] Carvalho M., Montero D., Domenici P., Afonso J.M., Izquierdo M. (2022). Dietary novel oils modulate neural function and preserve locomotor response in gilthead sea bream (*Sparus aurata*) juveniles by regulating synthesis and contents of fatty acids in brain. Aquaculture.

[55] Péron M., Soudant P., Le Grand F., Mazurais D., Simon V., Lefrançois C., Vagner M. (2025). Dietary DHA limitation did not affect swimming and metabolic performance, but reduced growth in wild European sea bass. Biochimie.

[56] Marques A., Canada P., Costa C., Basto A., Piloto F., Salgado M.A., Abreu H., Dias J., Valente L.M. (2023). Replacement of fish oil by alternative n-3 LC-PUFA rich lipid sources in diets for European sea bass (*Dicentrarchus labrax*). Front. Mar. Sci..

[57] Hundal B.K., Lutfi E., Sigholt T., Rosenlund G., Liland N.S., Glencross B., Sissener N.H. (2022). A piece of the puzzle—Possible mechanisms for why low dietary EPA and DHA cause hepatic lipid accumulation in Atlantic Salmon (*Salmo salar*). Metabolites.

[58] Bou M., Berge G.M., Baeverfjord G., Sigholt T., Østbye T.-K., Ruyter B. (2017). Low levels of very-long-chain n-3 PUFA in Atlantic salmon (*Salmo salar*) diet reduce fish robustness under challenging conditions in sea cages. J. Nutr. Sci..

[59] Sanden M., Liland N.S., Sæle Ø., Rosenlund G., Du S., Torstensen B.E., Stubhaug I., Ruyter B., Sissener N.H. (2016). Minor lipid metabolic perturbations in the liver of Atlantic salmon (*Salmo salar* L.) caused by suboptimal dietary content of nutrients from fish oil. Fish Physiol. Biochem..

[60] Subaramaniyam U., Allimuthu R.S., Vappu S., Ramalingam D., Balan R., Paital B., Panda N., Rath P.K., Ramalingam N., Sahoo D.K. (2023). Effects of microplastics, pesticides and nano-materials on fish health, oxidative stress and antioxidant defense mechanism. Front. Physiol..

[61] Bakiu R., Piva E., Pacchini S., Santovito G. (2024). Antioxidant systems in extremophile marine fish species. J. Mar. Sci. Eng..

[62] Wu D., Li J., Fan Z., Wang L., Zheng X. (2022). Resveratrol ameliorates oxidative stress, inflammatory response and lipid metabolism in common carp (*Cyprinus carpio*) fed with high-fat diet. Front. Immunol..

[63] Fan Z., Ma K., Wang Y., Wang L., Zhang Y., Li C., Li J., Wu D., Li J., Li Z. (2024). Liver transcriptome and physiological analyses preliminarily revealed the adaptation mechanisms of Amur grayling (*Thymallus arcticus grubei*, Dybowski, 1869) fry for dietary lipid nutrition. Front. Vet. Sci..

[64] Selvam C., Philip A.J.P., Lutfi E., Sigholt T., Norberg B., Bæverfjord G., Rosenlund G., Ruyter B., Sissener N.H. (2022). Long-term feeding of Atlantic salmon with varying levels of dietary EPA + DHA alters the mineral status but does not affect the stress responses after mechanical delousing stress. Br. J. Nutr..

[65] Seibel H., Baßmann B., Rebl A. (2021). Blood will tell: What hematological analyses can reveal about fish welfare. Front. Vet. Sci..

[66] Barton B.A. (2002). Stress in fishes: A diversity of responses with particular reference to changes in circulating corticosteroids. Integr. Comp. Biol..

[67] Yang L., Liu M., Zhao M., Zhi S., Zhang W., Qu L., Xiong J., Yan X., Qin C., Nie G. (2023). Dietary Bile Acid Supplementation Could Regulate the Glucose, Lipid Metabolism, and Microbiota of Common Carp (*Cyprinus carpio* L.) Fed with a High-Lipid Diet. Aquac. Nutr..

[68] Gong Y., Lu Q., Liu Y., Xi L., Zhang Z., Liu H., Jin J., Yang Y., Zhu X., Xie S. (2022). Dietary berberine alleviates high carbohydrate diet-induced intestinal damages and improves lipid metabolism in largemouth bass (*Micropterus salmoides*). Front. Nutr..

[69] Taylor R.S., Ruiz Daniels R., Dobie R., Naseer S., Clark T.C., Henderson N.C., Boudinot P., Martin S.A., Macqueen D.J. (2022). Single cell transcriptomics of Atlantic salmon (*Salmo salar* L.) liver reveals cellular heterogeneity and immunological responses to challenge by *Aeromonas salmonicida*. Front. Immunol..

[70] Roques S., Deborde C., Richard N., Skiba-Cassy S., Moing A., Fauconneau B. (2020). Metabolomics and fish nutrition: A review in the context of sustainable feed development. Rev. Aquac..

[71] Yang Z., Zhu H., Huang X., Wang A., Xie D. (2022). Molecular characterization, tissue distribution profile, and nutritional regulation of ACSL gene family in golden pompano (*Trachinotus ovatus*). Int. J. Mol. Sci..

[72] Xie D., He Z., Dong Y., Gong Z., Nie G., Li Y. (2020). Molecular Cloning, Characterization, and expression regulation of acyl-CoA synthetase 6 gene and promoter in common carp *Cyprinus carpio*. Int. J. Mol. Sci..

[73] Long T., Hassan A., Thompson B.M., McDonald J.G., Wang J., Li X. (2019). Structural basis for human sterol isomerase in cholesterol biosynthesis and multidrug recognition. Nat. Commun..

[74] Wang Y., Lan T., Zhou C., Zhang Q., Liu P. (2025). LRP8-dependent cholesterol metabolism modulates mTORC1 signaling and apoptotic pathways in multiple myeloma. Cell Death Dis..

[75] Subczynski W.K., Pasenkiewicz-Gierula M., Widomska J., Mainali L., Raguz M. (2017). High cholesterol/low cholesterol: Effects in biological membranes: A review. Cell Biochem. Biophys..

[76] Bibi H., Ahmad R., Rahman F., Maqbool S., Naeem M., Efthymiou S., Houlden H. (2025). Molecular and computational analysis of a novel pathogenic variant in emopamil-binding protein (EBP) involved in cholesterol biosynthetic pathway causing a rare male EBP disorder with neurologic defects (MEND syndrome). Mol. Biol. Rep..

[77] Wu G.F., Luo Z.G., Gao K., Ren Y., Shen C., Ying X.R. (2025). LRP8 Regulates Lipid Metabolism to Stimulate Malignant Progression and Cisplatin Resistance in Bladder Cancer. Kaohsiung J. Med. Sci..

[78] Xia R., Peng H.-F., Zhang X., Zhang H.-S. (2024). Comprehensive review of amino acid transporters as therapeutic targets. Int. J. Biol. Macromol..

[79] Menchini R.J., Chaudhry F.A. (2019). Multifaceted regulation of the system A transporter Slc38a2 suggests nanoscale regulation of amino acid metabolism and cellular signaling. Neuropharmacology.

[80] Hellsten S.V., Tripathi R., Ceder M.M., Fredriksson R. (2018). Nutritional stress induced by amino acid starvation results in changes for Slc38 transporters in immortalized hypothalamic neuronal cells and primary cortex cells. Front. Mol. Biosci..

[81] Calo J., Comesana S., Fernandez-Maestu C., Blanco A.M., Morais S., Soengas J.L. (2024). Impact of feeding diets with enhanced vegetable protein content and presence of umami taste-stimulating additive on gastrointestinal amino acid sensing and feed intake regulation in rainbow trout. Aquaculture.

[82] Bavia L., Santiesteban-Lores L.E., Carneiro M.C., Prodocimo M.M. (2022). Advances in the complement system of a teleost fish, *Oreochromis niloticus*. Fish Shellfish. Immunol..

[83] Bai H., Mu L., Qiu L., Chen N., Li J., Zeng Q., Yin X., Ye J. (2022). Complement C3 regulates inflammatory response and monocyte/macrophage phagocytosis of *Streptococcus agalactiae* in a teleost fish. Int. J. Mol. Sci..

[84] Zhang Y., Su J. (2023). Interleukin-2 family cytokines: An overview of genes, expression, signaling and functional roles in teleost. Dev. Comp. Immunol..

[85] Rodríguez-Viera L., Caderno A., Martinez R., Martinez-Rodríguez G., Oliva M., Perera E., Mancera J.M., Martos-Sitcha J.A. (2025). The Ghrelin Analog GHRP-6, Delivered Through Aquafeeds, Modulates the Endocrine and Immune Responses of *Sparus aurata* Following IFA Treatment. Biology.

[86] Wu F., Wang Z., Yang G., Jian J., Lu Y. (2022). Molecular characterization and expression analysis of interleukin-15 (IL-15) genes in orange-spotted grouper (*Epinephelus coioides*) in response to Vibrio harveyi challenge. Fish Shellfish. Immunol..

[87] Ferretti E., Corcione A., Pistoia V. (2017). The IL-31/IL-31 receptor axis: General features and role in tumor microenvironment. J. Leukoc. Biol..

[88] Huang I.-H., Chung W.-H., Wu P.-C., Chen C.-B. (2022). JAK–STAT signaling pathway in the pathogenesis of atopic dermatitis: An updated review. Front. Immunol..

[89] Van Loo G., Bertrand M.J. (2023). Death by TNF: A road to inflammation. Nat. Rev. Immunol..

[90] Jin M., Shen Y., Pan T., Zhu T., Li X., Xu F., Betancor M.B., Jiao L., Tocher D.R., Zhou Q. (2021). Dietary betaine mitigates hepatic steatosis and inflammation induced by a high-fat-diet by modulating the Sirt1/Srebp-1/Pparɑ pathway in juvenile black seabream (*Acanthopagrus schlegelii*). Front. Immunol..

[91] Ahmed H., Bakry K.A., Abdeen A., El Bahgy H.E., Abdo M., Imbrea F., Fericean L., Elshemy M.A., Ibrahim S.F., Shukry M. (2023). The involvement of antioxidant, stress, and immune-related genes in the responsive mechanisms of common carp (*Cyprinus carpio*) to hypersalinity exposure. Front. Mar. Sci..

[92] Li Y., Zhou C., Zhang Y., Zhao X. (2024). Effects of Heat Stress on the Muscle Meat Quality of Rainbow Trout. Fishes.

[93] Zou J., Secombes C.J. (2016). The function of fish cytokines. Biology.

[94] Qi P., Xie C., Guo B., Wu C. (2016). Dissecting the role of transforming growth factor-β1 in topmouth culter immunobiological activity: A fundamental functional analysis. Sci. Rep..

